# No Genetic Causal Association Between Periodontitis and Arthritis: A Bidirectional Two-Sample Mendelian Randomization Analysis

**DOI:** 10.3389/fimmu.2022.808832

**Published:** 2022-01-26

**Authors:** Kang-Jia Yin, Ji-Xiang Huang, Peng Wang, Xiao-Ke Yang, Sha-Sha Tao, Hong-Miao Li, Jing Ni, Hai-Feng Pan

**Affiliations:** ^1^ Department of Epidemiology and Biostatistics, School of Public Health, Anhui Medical University, Hefei, China; ^2^ Inflammation and Immune Mediated Diseases Laboratory of Anhui Province, Hefei, China; ^3^ Department of Rheumatology and Immunology, The First Affiliated Hospital of Anhui Medical University, Hefei, China

**Keywords:** periodontitis, osteoarthritis, rheumatoid arthritis, Mendelian randomization, causal relationship

## Abstract

**Objectives:**

Periodontitis (PD) has been linked to arthritis in previous epidemiological observational studies; however, the results are inconclusive. It remains unclear whether the association between PD and arthritis is causal. The purpose of this study was to investigate the causal association of PD with arthritis, including rheumatoid arthritis (RA) and osteoarthritis (OA).

**Methods:**

We performed a two-sample bidirectional Mendelian randomization (MR) analysis using publicly released genome-wide association studies (GWAS) statistics. The inverse-variance weighted (IVW) method was used as the primary analysis. We applied four complementary methods, including weighted median, weighted mode, MR-Egger regression and MR pleiotropy residual sum and outlier (MR-PRESSO) to detect and correct for the effect of horizontal pleiotropy.

**Results:**

Genetically determined PD did not have a causal effect on OA (OR = 1.06, 95% CI: 0.99-1.15, *P* = 0.09) and RA (OR = 0.99, 95% CI: 0.87-1.13, *P* = 0.89). Furthermore, we did not find a significant causal effect of arthritis on PD in the reverse MR analysis. The results of MR-Egger regression, Weighted Median, and Weighted Mode methods were consistent with those of the IVW method. Horizontal pleiotropy was unlikely to distort the causal estimates according to the sensitivity analysis.

**Conclusions:**

Our MR analysis reveals non-causal association of PD with arthritis, despite observational studies reporting an association between PD and arthritis.

## Introduction

Arthritis is an acute or chronic inflammation of the joints that is typically accompanied by pain and structural damage (1). The most frequent types of arthritis are rheumatoid arthritis (RA) and osteoarthritis (OA), and they exhibit phenotypic similarities and overlapping molecular characteristics in spite of different pathogeneses (2). RA is a multi-systemic autoimmune disease characterized by inflammation of the synovial membrane which imposes a tremendous burden on the individual and society (3). RA has a prevalence of 0.5% to 1% and the interaction of genetic predisposition, environmental and hormonal factors is considered as the leading causes of RA (4). OA is a whole-joint disease characterized by articular cartilage loss and alterations in the subchondral bone (5). Emerging evidence suggested that systemic inflammation may play a role in the development of OA and synovium inflammation is implicated in the pathogenesis of OA (6). OA is becoming more prevalent with the combined effects of ageing and increasing obesity, and approximately 250 million individuals worldwide are currently affected by this disease (7).

Periodontitis (PD) is an inflammatory illness of the oral cavity caused by bacterial infection of the periodontal tissue, leading to the resorption of alveolar bone and tooth loss eventually (8). PD is one of the most common chronic inflammatory diseases which affects 46% of the United States population according to the National Health and Nutrition Examination Survey and severe PD affect 10-15% of the population globally (9, 10). PD can be classified as the Grade C periodontitis, known as aggressive periodontitis (AgP) in 1999 classification and a slowly progressive form of periodontitis which is formerly known as chronic periodontitis (CP) (11). AgP is a severe and early-onset form of PD with a high progression rate and a prevalence of ~0.1% (12). CP is the more frequent phenotype, affecting an older population with a slow disease progression. PD is reported to be associated with several diseases, including stroke (13), cardiovascular disease (14), and pneumonia (15).

It has long been recognized that there are significant parallels between PD and arthritis. Inflammatory cytokines such as tumor necrosis factor (TNF) and interleukin-6 (IL-6), as well as initial activation of the innate immune system play a crucial role in both disorders (16). Moreover, abundant evidence from observational studies demonstrated that there is an association between PD and arthritis. Results from several case-control studies underlined that the prevalence of PD in RA patients is higher than in healthy controls (17, 18). Kim et al. reported that PD was linked to the occurrence and the severity of radiographic knee OA (19). However, the causal relationship between PD and arthritis has yet to be established, which is extremely important and could improve our present understanding of PD and arthritis pathogeneses.

Reverse causation, measurement error, and underlying bias are all intrinsic limitations of observational studies. Therefore, we employed Mendelian randomization (MR) analysis, which incorporates germline genetic variants as instrumental variables (IVs) for exposure, to study the causal relationship between exposure phenotype and outcome phenotype (20). Three assumptions of MR must be met in order to obtain impartial results: (a) the genetic IVs should have a strong link to the exposure; (b) the genetic IVs are not associated with confounders linked to the chosen exposure and outcome; (c) the genetic IVs influence the outcome only through the exposures and not *via* other biological pathways (21). Here, we conducted two-sample MR analysis to evaluate the causal connection between PD and the risk of arthritis. Moreover, we also conducted reverse MR to look at the bidirectional causal effect of arthritis on PD.

## Methods

### Data Sources

Summary statistics for PD were obtained from the latest meta-analysis of GWAS of the Gene-Lifestyle Interaction in the Dental Endpoints (GLIDE) Consortium, with the largest sample size to date involving 17,353 clinically diagnosed cases and 28,210 controls (22). There were 12,289 cases and 22,326 controls with European descent incorporated after excluding the Hispanic Community Health Study/Study of Latinos (HCHS/SOL) in the GLIDE consortium, as the participants were recruited from Hispanic and Latino communities in the USA. SNPs associated with AgP were derived from the discovery stage of the GWAS in a sample of 851 cases and 6,580 controls from Germany and The Netherlands (23). Thirteen lead SNPs outperformed the predetermined selection criterion of the GWAS in the discovery stage and were proposed as being associated with AgP with a significant *p*-value < 1×10^-5^. SNPs associated with CP were sourced from the genome-wide association meta-analysis of the Study of Health in Pomerania (SHIP) and SHIP-TREND cohorts, including 1,817 cases and 2,215 controls, where twenty-two SNPs with *p*-value < 1×10^-5^ were deemed to be related to CP and were used as IVs (24).

Genetic statistics for arthritis were derived from two separate GWAS for RA and OA. Summary statistics for RA were derived from a meta-analysis of GWAS, which included 14,361 cases and 43,923 controls of European ancestry (25). Summary-level statistics for OA were obtained from the discovery cohort of the GWAS, which included 10,083 hospital-diagnosed cases and 40,425 controls and were conducted using genotyping data from the UK Biobank collection (26). In this GWAS, OA at any site was clinically diagnosed based on ICD10 hospital-record codes. To eliminate population stratification bias, all SNPs and their accompanying summary data were retrieved from studies that solely included populations of European ancestry.

### Selection of the Genetic Instruments

To filter eligible genetic IVs that fulfill the three core MR assumptions, we performed a set of quality control techniques. Firstly, we selected independent SNPs that were strongly associated with RA with *p*-value less than 5×10^-8^. Since there were no SNPs with *p*-value less than 5×10^-8^ for PD and OA, we broadened the threshold to 1×10^-5^ to select eligible instrumental variables. Secondly, to exclude SNPs that were in strong linkage disequilibrium (LD), we performed the clumping procedure with *R^2^* < 0.001 and a window size = 10,000 kb with the European ancestral individuals from the 1000 Genomes Project (27). SNP having a lower *p* value would be kept among SNP pairings with an LD *R^2^* greater than the stated threshold. Thirdly, SNPs with minor allele frequency (MAF) of less than 0.01 were eliminated as well. Fourthly, when the targeted SNPs were not found in the outcome GWAS, proxy SNPs having high LD (*R* ^2^ >0.8) with the target SNPs would be screened for instead. Finally, to guarantee that the effect alleles belong to the same allele, we harmonized the exposure and outcome datasets to eliminate ambiguous SNPs with non-concordant alleles and SNPs with intermediate allele frequencies. We utilized these carefully chosen SNPs as the final genetic IVs for the subsequent MR analysis.

Furthermore, we calculated the *F* statistics for each SNP solely and cumulatively by the following equation: F=R^2^×(N - 2)/(1 - R^2^). R^2^ denotes the variance of exposure explained by each IV. IVs with *F* statistics of less than ten were considered weak instruments and would be excluded for MR analysis (28).

### Statistical Analysis

In this study, we applied multiple complementary approaches, including the inverse variance weighted (IVW), the MR-Egger regression, the Weighted Median, and the Weighted Mode methods, to estimate the causal effects of exposures on outcomes. The IVW method was used as the major analysis method. We calculated the statistical power using the mRnd website (https://shiny.cnsgenomics.com/mRnd/) (29).

The IVW method was primarily employed for fundamental causal estimates, which would provide the most precise results when all selected SNPs were valid IVs. The IVW method calculates a weighted average of Wald ratio estimates (30). Under the assumption of Instrument Strength Independent of Direct Effect (InSIDE), the MR-Egger regression executes a weighted linear regression and yields a consistent causal estimate, even though the genetic IVs are all invalid (31). However, it exhibits low precision and is susceptible to outlying genetic variants. The Weighted Median regression method, which does not demand the InSIDE hypothesis, calculates a weighted median of the Wald ratio estimates and is robust to horizontal pleiotropic bias (32). It is confirmed that the Weighted Median method has some advantages over the MR-Egger regression, as it provides lower type I error and higher causal estimate power. Finally, the Weighted Mode method estimates the causal effect of the subset with the largest number of SNPs by clustering the SNPs into subsets resting on the resemblance of causal effects (33).

### Pleiotropy and Sensitivity Analysis

We conducted the MR-Egger regression to evaluate the possibility of horizontal pleiotropy. The average pleiotropic effect of the IVs is indicated by the intercept term of MR-Egger regression (31). The asymmetry of the funnel plot can also be considered as an indicator of horizontal pleiotropy (34). The MR Pleiotropy REsidual Sum and Outlier (MR-PRESSO) test was additionally conducted to evaluate whether pleiotropy was present (35). Its functions include detecting horizontal pleiotropy, correcting for horizontal pleiotropy by removing outlier, and determining whether there are substantial variations in the causal effects before and after outlier removal. To find heterogeneity, we employed the IVW approach and MR-Egger regression, the heterogeneities were quantified by Cochran’s Q statistic. In addition, we utilized the leave-one-out analysis to detect the robustness and consistency of the results.

Multiple comparisons were corrected using the Bonferroni method, and a *p*-value less than 0.006 (0.05/8) indicated compelling evidence of causal relations. All analysis were carried out using packages “TwoSampleMR” (34) and “MRPRESSO” in R version 4.0.3.

## Results

### Causal Effects of PD and Subtypes on Arthritis

We incorporated 18, 13, and 22 independent SNPs with significant *p*-value less than 1×10^-5^ as IV SNPs for PD, AgP and CP. However, two PD-associated SNPs, one AgP-associated SNP, and two CP-associated SNPs were not available in the summary statistic of RA. Only one PD-associated SNP of the five unavailable SNPs was replaced by proxy SNP rs4969455 with R^2^ > 0.8, and none of the genetic variants was palindromic with intermediate allele frequencies. Of the IVs for PD and subtypes mentioned above, one CP-associated SNP was not available in the summary statistic of OA and so was excluded as there was no suitable proxy SNP. The *F*-statistic values were all more than 10, with average *F*-statistic values of 21.22, 21.69, and 21.52 for PD, AgP and CP. Detailed information of IVs for PD and subtypes was listed in **Supplementary Table 1**. The variance explained by these IVs were 1% for PD, 3.8% for AgP and 12% for CP. Our MR analysis yield sufficient power (above 90% to detect an OR of 1.20) to find moderate relationships between AgP, CP and arthritis, but lower power in the study of CP’s effect on arthritis (51% power to detect an OR of 1.20).

**Figure 1:**
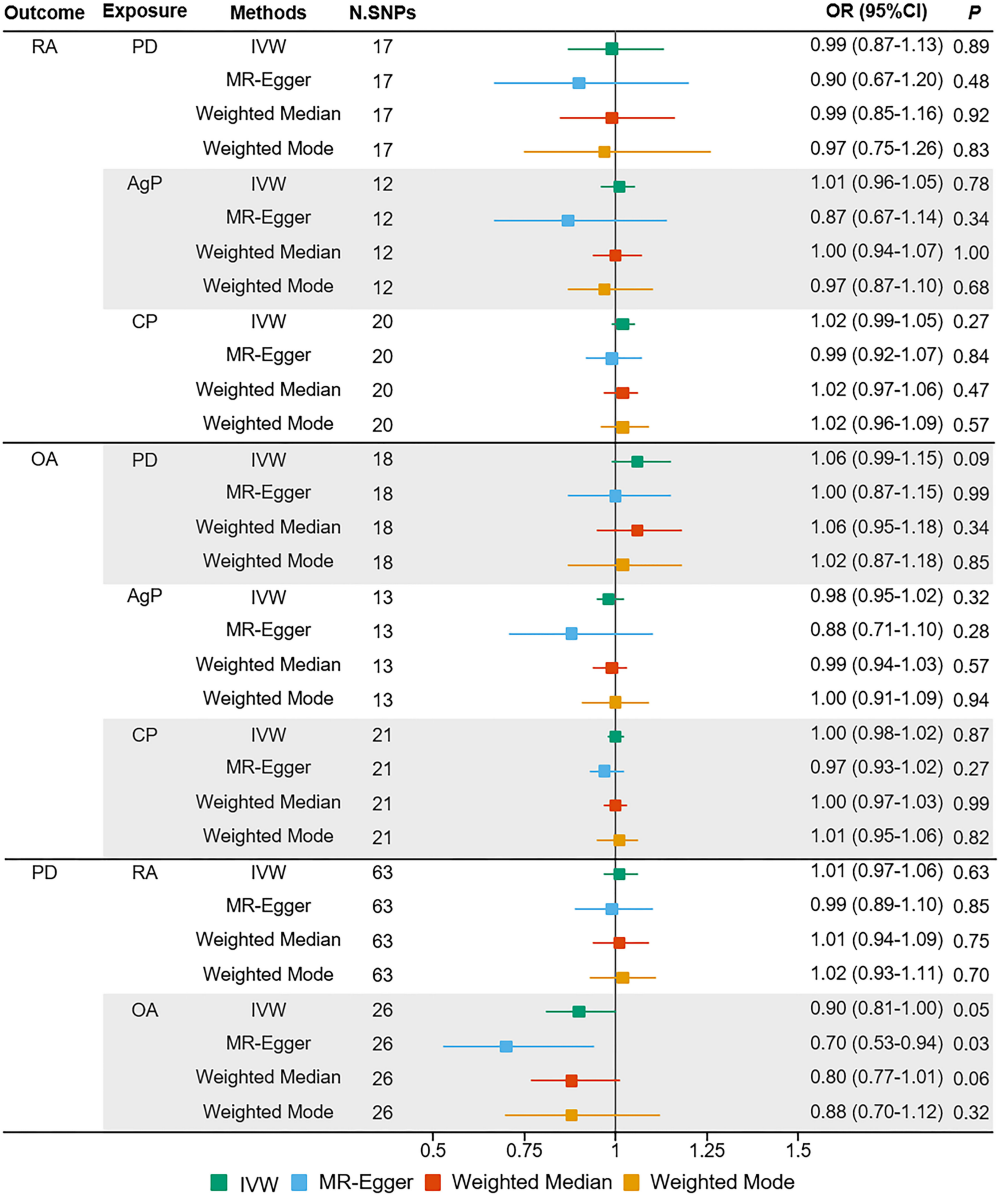
Estimated causal effects between PD and arthritis using different MR methods. AgP, aggressive periodontitis; CI, confidence interval; CP, chronic periodontitis; IVW, inverse variance weighted; N.SNPs, number of SNPs used in MR; OR, odds ratio; PD, periodontitis; RA, rheumatoid arthritis; OA, osteoarthritis.

**Figure 2:**
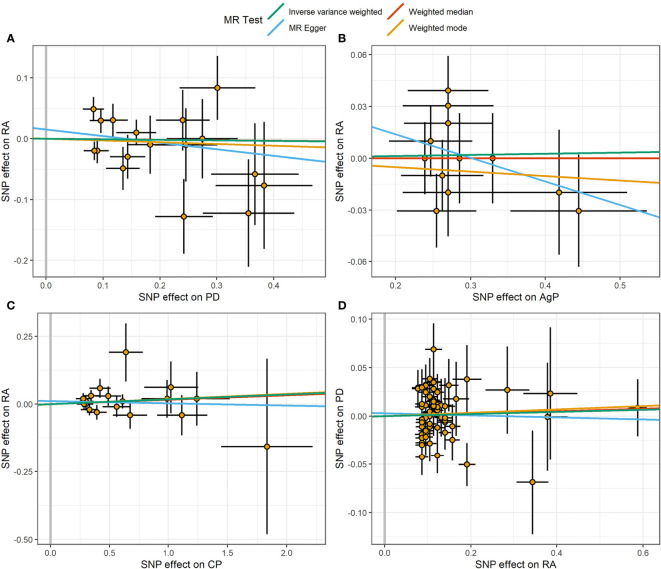
Scatter plot of the causal relationships between RA and PD using different MR methods. **(A)** Causal estimates for PD on RA. **(B)** Causal estimates for AgP on RA. **(C)** Causal estimates for CP on RA. **(D)** Causal estimates for RA on PD. The slope of each line corresponding to the causal estimates for each method. Individual SNP-effect on the outcome (point and vertical line) against its effect on the exposure (point and horizontal line) was delineated in the background.

**Figure 3:**
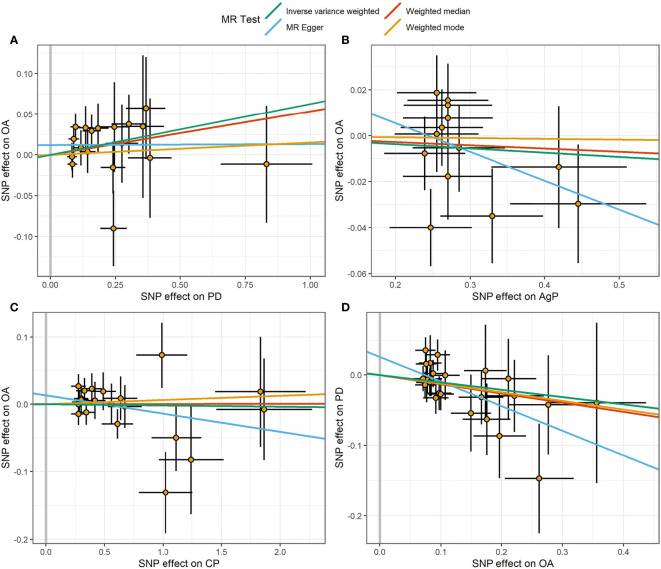
Scatter plot of the causal relationships between OA and PD using different MR methods. **(A)** Causal estimates for PD on OA. **(B)** Causal estimates for AgP on OA. **(C)** Causal estimates for CP on OA. **(D)** Causal estimates for OA on PD. The slope of each line corresponding to the causal estimates for each method. Individual SNP-effect on the outcome (point and vertical line) against its effect on the exposure (point and horizontal line) was delineated in the background.

The MR estimates of different methods were presented in **Figure 1**. Overall, there were no causal associations between genetically predicted PD and arthritis risk. The primary results of IVW showed that an increase in the risk of having PD was not statistically related with an increased risk of having arthritis (RA: OR=0.99, 95% CI: 0.87-1.13, *P* = 0.89; OA: OR=1.06, 95% CI: 0.99-1.15, *P* = 0.09). In addition, the MR-Egger, the Weighted Median, and the Weighted Mode methods showed consistent results. Furthermore, the MR estimates showed that the causal effects of AgP and CP on arthritis were the same as those of PD. The scatter plot for effect sizes of SNPs for PD and its subtypes and those for arthritis were shown in **Figures 2**, **3**. There was no heterogeneity between the individual SNP according to the heterogeneity test. Horizontal pleiotropy was unlikely to skew the causality of PD with arthritis, according to the results of the MR-Egger regression and MR-PRESSO global test (**Table 1** and **Supplementary Table 4**). Leave-one-out analysis indicated that the causal estimates of PD and subtypes were not driven by any single SNP. The leave-one-out analysis plots, forest plots, and funnel plots were shown in **Supplementary Figures S1**–**S6**.

**Table 1:** MR estimates of assessing the causal association between PD and arthritis..

Exposure	Outcome	N. SNPs	F-statistic	IVW				MR-Egger			
				OR (95%CI)	*P*	Q statistic	Q_pval	OR (95%CI)	*P*	Intercept	*P*_inter_
PD	RA	17	21.22	0.99 (0.87, 1.13)	0.89	25.12	0.07	0.90 (0.67, 1.20)	0.48	0.015	0.47
	OA	18		1.06 (0.99, 1.15)	0.09	14.90	0.60	1.00 (0.87, 1.15)	0.99	0.012	0.30
AgP	RA	12	21.69	1.01 (0.96, 1.05)	0.78	10.92	0.45	0.87 (0.67, 1.14)	0.34	0.041	0.31
	OA	13		0.98 (0.95, 1.02)	0.32	13.35	0.34	0.88 (0.71, 1.10)	0.28	0.031	0.35
CP	RA	20	21.52	1.02 (0.99, 1.05)	0.27	13.86	0.79	0.99 (0.92, 1.07)	0.84	0.011	0.50
	OA	21		1.00 (0.98, 1.02)	0.87	17.41	0.63	0.97 (0.93, 1.02)	0.27	0.013	0.24
RA	PD	63	56.98	1.01 (0.97, 1.06)	0.63	73.98	0.14	0.99 (0.89, 1.10)	0.85	0.003	0.66
OA	PD	26	21.04	0.90 (0.81, 1.00)	0.05	17.61	0.86	0.70 (0.53, 0.94)	0.03	0.026	0.09

AgP, aggressive periodontitis; CI, confidence interval; CP, chronic periodontitis; IVW, inverse variance weighted; N. SNPs, number of SNPs used in MR; OA, osteoarthritis; OR, odds ratio; PD, periodontitis; RA, rheumatoid arthritis.

P_inter,_ P value for intercept test of multivariable MR Egger.

### Causal Effects of Arthritis on PD

We incorporated 64 and 26 independent SNPs as IVs for RA and OA. One RA-associated SNP was not available in the summary statistic of PD and so was excluded as there was no suitable proxy SNP to replace. The *F*-statistic values were all more than 10, with average *F*-statistic values of 56.98 and 21.04 for RA and OA. Detailed information of instrumental variables for arthritis was listed in **Supplementary Tables 2**–**3**. The variance explained by these IVs were 5.7% for RA and 1.1% for OA. Our MR analysis yield sufficient power (above 90% to detect an OR of 1.20) to find moderate association of RA and PD, but lower power in the study of the effect of OA on PD (42% power to detect an OR of 1.20). Based on the IVW method, there were no indication of causal link between arthritis and the risk of PD (RA: OR=1.01, 95% CI: 0.97-1.06, *P* = 0.63; OA: OR=0.90, 95% CI: 0.81-1.00, *P* = 0.05) (**Figure 1**). No significant evidence of horizontal pleiotropy was observed for IVs of arthritis (**Table 1**).

## Discussion

This was the first study to investigate the bidirectional causal association between PD and arthritis by conducting multiple complementary MR methods. Our two-sample MR analysis did not observe evidence supporting that genetically predicted PD was associated with arthritis in individuals from European descent. The reverse MR analysis similarly found no evidence that genetic liability to arthritis was related to PD.

Previous epidemiological studies that noted a link between PD and arthritis have been undertaken. Overall, a recent meta-analysis reported that the risk of developing PD in patients with RA was 1.13 times higher than in healthy controls (RR: 1.13; 95%CI: 1.04, 1.23; *P*= 0.006) (36). Several case-control studies have found that RA patients have a higher prevalence of PD than healthy controls (37–39). Furthermore, a latest meta-analysis comprising of 6 case-controls studies has reported that PD was associated with substantially increased RA disease activity, with an average of 0.74 DAS28 score increase compared to non-PD patients (95%CI: 0.25, 1.24; *P*<0.001) (40). A case-control study that included participants from the Korean National Health and Nutrition Examination Survey demonstrated that the incidence and severity of radiographic knee OA were linked to PD (19). In addition, PD was also reported to be associated with ankylosing spondylitis (AS) and axial Spondyloarthritis (AxSpA) in several case-control studies (41, 42).

However, not every study has drawn the same conclusion of the association between arthritis and PD (43, 44). A case-control study conducted by the Swedish Epidemiological Investigation of RA (EIRA) found no evidence of an increased prevalence of PD in established RA patients compared to healthy controls, and no differences on the basis of anti-citrullinated protein antibody (ACPA) or rheumatoid factor (RF) status among RA patients (43). Therefore, it is difficult to sustain a causal relationship between PD and RA based purely on observational studies.

There are several possible reasons that may explain the association between PD and arthritis in observational studies. Firstly, the oral microbiota may be a shared risk for both RA and PD. It was reported that the periodontal pathogen *Porphyromonas gingivalis* (*P. gingivalis*), which is gram-negative anaerobe bacteria and is characterized by the expression of peptidylarginine deiminase (PAD) and the process of citrullination, may interpret the biological intersection between PD and RA (45). PAD enzyme mediates the development of RA by catalyzing citrullination, and results in the generation of ACPA, which is generally known as diagnostic biomarkers for RA patients (46, 47). Moreover, it has been reported that an increased titer of antibody against *P.gingivalis* was detected in the serum and synovial fluid of RA patients (48). Periodontal pathogen *Enterococcus faecalis* was found in knee joint tissues of OA patients undergoing knee replacement and arthroplasty (49). More recently, the oral pathogen *Aggregatibacter actinomycetemcomitans (Aa)* has been suggested to play a role in RA. It could secrete leukotoxin A (LtxA) and induce hypercitrullination at the neutrophil level. This mechanism leads to the hypercitrullinated autoantigen release and immunological response finally (50). Secondly, gut microbiota has been considered as an environmental factor for RA development and progression. Sato et al. found that the modification of gut microbiota induced by *P.gingivalis* is associated with aggravated collagen-induced arthritis (CIA), with increased IL-17 levels in the serum, increased Th17 cell proportions in the lymphocytes. Those results point to a unique role of *P.gingivalis* in the link between PD and RA by affecting the gut microbiota composition (51). Thirdly, some genetic variations may be linked to the increased susceptibility to both PD and RA, with the HLA-DRB1 alleles that code shared epitope (SE) being the common genetic factor. Genetic factors account for 50% of the risk to develop RA and the SE-coding HLA-DRB1 gene account for more than 80% of joint destruction susceptibility (52). Marotte et al. identified a significant association between SE positivity and bone destruction in periodontal site with SE+ patients having a 2.2 times greater risk to have periodontal destruction compared to SE- (53). Fourthly, the common risk factor of smoking may provide a possible confounder in the association study between PD and arthritis. Smoking was found to be a risk factor for both arthritis (54, 55) and PD (56) in previous MR studies.

The current bidirectional MR study has several strengths. First, this study further obtained the summary statistics of two subtypes of PD to calculate the causal association with arthritis. Because of the early-onset and lack of risk factors such as long-term smoking or diabetes, it is thought that genetic liability plays a major role in the development and progression of AgP (23). Negative results from the causal association of MR analysis between AgP and arthritis further confirmed the causal link between PD and arthritis. Another strength is that the bidirectional analysis guaranteed the inference of causality between PD and arthritis in both directions. Meanwhile, there are several limitations in this study. First, the study population included in the MR analysis was European ancestry. Hence, whether the results can be representative of the whole population remains to be verified. Second, there were likely overlapping participants in the exposure and outcome studies, but it is difficult to estimate the degree of sample overlap. Fortunately, the usage of strong instruments (e.g., *F* statistic much greater than 10) in this study should minimize potential bias from sample overlap (57). Third, SNPs used as genetic instruments were weakly associated with PD subtypes and OA, with a threshold of *P*-value < 1×10^-5^. And limited SNPs were selected as IVs, which may only explain a small proportion of the variation of exposure and affect the statistical power of the causal estimates.

In conclusion, the results of our study did not demonstrate a causal effect of genetically predicted PD and subtypes on arthritis, neither did genetically predicted arthritis on PD. Updated MR analysis based on larger scale GWAS summary data and more genetic instruments is required to verify the results of this study.

## Data Availability Statement

The summary statistics used and/or analyzed in the current study are available from the corresponding authors on reasonable request.

## Author Contributions

H-FP and JN conceived the present idea and were responsible for the design of the study. K-JY, J-XH, and PW access all the data in the study and took responsibility for the accuracy of the data analysis. K-JY performed the statistical analysis and manuscript writing. All authors were involved in the writing and revision of the article, and all authors approved the submitted version to be published.

## Funding

This study was funded by grants from Anhui Provincial Natural Science Foundation (2108085QH361, 2108085Y26), the National Natural Science Foundation of China (81872687, 82103932), Research Fund of Anhui Institute of Translational Medicine (2021zhyx-B04), and Nature Science Foundation of Anhui Medical University (2020xkj011).

## Conflict of Interest

The authors declare that the research was conducted in the absence of any commercial or financial relationships that could be construed as a potential conflict of interest.

## Publisher’s Note

All claims expressed in this article are solely those of the authors and do not necessarily represent those of their affiliated organizations, or those of the publisher, the editors and the reviewers. Any product that may be evaluated in this article, or claim that may be made by its manufacturer, is not guaranteed or endorsed by the publisher.
