# Supplementary material for: No Genetic Causal Association Between Periodontitis and Arthritis: A Bidirectional Two-Sample Mendelian Randomization Analysis
Source: Front Immunol. 2022 Jan 26;13:808832. doi: 10.3389/fimmu.2022.808832 (PMC8825874; doi:10.3389/fimmu.2022.808832)
Supplement: Supplementary file 1 [file DataSheet_1.docx]

Supplementary Materials

**Table S1.** Detailed information of instrumental variables utilized in the Mendelian Randomization analysis of periodontitis phenotypes on rheumatoid arthritis and osteoarthritis

| SNP | Chr | Pos | EA/OA | EAF | Association with PD | | | |  | Association with RA | | |  | Association with OA | | |
| --- | --- | --- | --- | --- | --- | --- | --- | --- | --- | --- | --- | --- | --- | --- | --- | --- |
|  |  |  |  |  | *β* | S.E. | *P*-value | *F*-statistic |  | *β* | S.E. | *P*-value |  | *β* | S.E. | *P*-  value |
| Instrumental variables of PD | | | | | | | | | | | | | | | | |
| rs10757466 | 9 | 24000258 | T/C | 0.14 | -0.117 | 0.026 | 9.26E-06 | 19.704 |  | -0.030 | 0.027 | 0.350 |  | -0.009 | 0.022 | 0.693 |
| rs118009719 | 8 | 86928036 | A/C | 0.04 | -0.275 | 0.062 | 9.24E-06 | 19.669 |  | 0.000 | 0.065 | 1.000 |  | -0.014 | 0.048 | 0.773 |
| rs13005050 | 2 | 52705571 | T/C | 0.88 | -0.143 | 0.031 | 3.76E-06 | 21.332 |  | 0.030 | 0.036 | 0.390 |  | -0.004 | 0.026 | 0.878 |
| rs148287804 | 2 | 43560590 | T/G | 0.98 | -0.383 | 0.085 | 6.22E-06 | 20.446 |  | 0.077 | 0.104 | 0.480 |  | 0.004 | 0.073 | 0.958 |
| rs151226594 | 11 | 64256137 | T/G | 0.97 | -0.367 | 0.077 | 1.75E-06 | 22.851 |  | 0.058 | 0.083 | 0.460 |  | -0.057 | 0.063 | 0.364 |
| rs184267209 | 12 | 94396361 | A/G | 0.02 | 0.246 | 0.055 | 8.91E-06 | 19.703 |  | -0.010 | 0.060 | 0.920 |  | 0.034 | 0.055 | 0.536 |
| rs186040223 | 7 | 64014696 | A/C | 0.85 | -0.158 | 0.035 | 5.66E-06 | 20.570 |  | -0.010 | 0.021 | 0.780 |  | -0.029 | 0.020 | 0.143 |
| rs1901299 | 18 | 22442179 | A/C | 0.07 | -0.090 | 0.020 | 8.63E-06 | 19.773 |  | 0.020 | 0.020 | 0.410 |  | -0.020 | 0.018 | 0.279 |
| rs190792824 | 20 | 42706632 | T/G | 0.04 | -0.301 | 0.066 | 5.75E-06 | 20.603 |  | -0.083 | 0.053 | 0.100 |  | -0.038 | 0.036 | 0.297 |
| rs28546695 | 18 | 49760987 | A/G | 0.38 | 0.083 | 0.019 | 8.21E-06 | 19.808 |  | 0.049 | 0.020 | 0.025 |  | -0.002 | 0.017 | 0.891 |
| rs2976950 | 8 | 8249082 | A/G | 0.60 | 0.096 | 0.020 | 7.99E-07 | 24.382 |  | 0.030 | 0.020 | 0.097 |  | 0.034 | 0.016 | 0.034 |
| rs4640758 | 5 | 7613236 | A/G | 0.36 | -0.085 | 0.019 | 7.28E-06 | 20.120 |  | 0.020 | 0.015 | 0.160 |  | 0.012 | 0.017 | 0.488 |
| rs4956201 | 4 | 109527782 | A/C | 0.06 | -0.241 | 0.047 | 3.89E-07 | 25.768 |  | -0.030 | 0.050 | 0.530 |  | 0.016 | 0.032 | 0.623 |
| rs4969455^*^ | 17 | 78785746 | T/C | 0.06 | -0.183 | 0.041 | 7.83E-06 | 19.981 |  | 0.010 | 0.048 | 0.890 |  | -0.033 | 0.031 | 0.285 |
| rs6816769 | 4 | 122216017 | T/C | 0.08 | -0.135 | 0.029 | 4.57E-06 | 21.020 |  | 0.049 | 0.035 | 0.160 |  | -0.033 | 0.027 | 0.224 |
| rs73155039^#^ | 7 | 136207654 | A/G | 0.99 | 0.832 | 0.176 | 2.22E-06 | 22.410 |  | NA | NA | NA |  | -0.011 | 0.072 | 0.874 |
| rs78422482 | 4 | 19970150 | A/G | 0.02 | 0.243 | 0.051 | 2.02E-06 | 22.614 |  | -0.128 | 0.062 | 0.045 |  | -0.090 | 0.046 | 0.049 |
| rs78982133 | 6 | 11688132 | A/G | 0.97 | -0.356 | 0.080 | 8.71E-06 | 19.773 |  | 0.122 | 0.089 | 0.170 |  | -0.035 | 0.088 | 0.694 |
| Instrumental variables of AgP | | | | | | | | | | | | | | | | |
| rs1002204 | 7 | 87141497 | C/A | 0.47 | 0.239 | 0.054 | 7.92E-06 | 19.584 |  | 0.000 | 0.021 | 0.820 |  | -0.008 | 0.016 | 0.627 |
| rs11382193 | 11 | 12230567 | A/AT | 0.63 | 0.255 | 0.056 | 5.76E-06 | 20.729 |  | 0.030 | 0.021 | 0.220 |  | 0.001 | 0.017 | 0.967 |
| rs11633566^#^ | 15 | 49394406 | G/A | 0.75 | 0.270 | 0.060 | 7.99E-06 | 20.245 |  | NA | NA | NA |  | -0.018 | 0.019 | 0.347 |
| rs1380780 | 3 | 163139348 | T/A | 0.72 | 0.270 | 0.059 | 4.27E-06 | 20.937 |  | 0.020 | 0.021 | 0.390 |  | 0.013 | 0.018 | 0.450 |
| rs17340482 | 1 | 27236578 | G/C | 0.90 | 0.445 | 0.091 | 9.99E-07 | 23.907 |  | -0.030 | 0.033 | 0.380 |  | -0.030 | 0.026 | 0.250 |
| rs2144815 | 14 | 95800526 | T/C | 0.17 | 0.329 | 0.069 | 1.53E-06 | 22.729 |  | 0.000 | 0.026 | 0.880 |  | -0.035 | 0.021 | 0.089 |
| rs2978951 | 8 | 6823295 | A/G | 0.41 | 0.270 | 0.054 | 4.49E-07 | 24.993 |  | 0.039 | 0.020 | 0.085 |  | 0.015 | 0.016 | 0.332 |
| rs4284742 | 19 | 52131733 | G/A | 0.76 | 0.285 | 0.062 | 4.66E-06 | 21.125 |  | 0.000 | 0.026 | 0.970 |  | -0.005 | 0.019 | 0.777 |
| rs4731202 | 7 | 79721630 | T/G | 0.66 | 0.247 | 0.055 | 8.54E-06 | 20.163 |  | 0.010 | 0.021 | 0.780 |  | -0.040 | 0.017 | 0.017 |
| rs6137428 | 20 | 2227689 | C/T | 0.90 | 0.419 | 0.091 | 3.92E-06 | 21.195 |  | -0.020 | 0.036 | 0.590 |  | -0.014 | 0.027 | 0.608 |
| rs6887423 | 5 | 36696501 | T/C | 0.42 | 0.255 | 0.053 | 1.42E-06 | 23.143 |  | -0.030 | 0.021 | 0.093 |  | 0.019 | 0.016 | 0.244 |
| rs744280 | 15 | 25960629 | C/T | 0.73 | 0.270 | 0.060 | 7.97E-06 | 20.245 |  | -0.020 | 0.026 | 0.470 |  | 0.008 | 0.018 | 0.674 |
| rs747804 | 9 | 35944775 | G/A | 0.64 | 0.262 | 0.055 | 1.65E-06 | 22.686 |  | -0.010 | 0.021 | 0.720 |  | 0.004 | 0.017 | 0.834 |
| Instrumental variables of CP | | | | | | | | | | | | | | | | |
| rs111571364 | 2 | 27859317 | T/G | 0.02 | 1.241 | 0.278 | 8.00E-06 | 19.928 |  | 0.020 | 0.099 | 0.880 |  | -0.082 | 0.081 | 0.302 |
| rs12048046 | 1 | 236912057 | T/C | 0.9 | 0.419 | 0.090 | 3.00E-06 | 21.805 |  | 0.058 | 0.035 | 0.110 |  | 0.005 | 0.028 | 0.846 |
| rs12587630 | 14 | 38066575 | A/G | 0.98 | 0.990 | 0.220 | 7.00E-06 | 20.183 |  | 0.020 | 0.070 | 0.790 |  | 0.072 | 0.049 | 0.134 |
| rs13145041^#^ | 4 | 190538063 | A/C | 0.78 | 0.501 | 0.101 | 7.00E-07 | 24.603 |  | NA | NA | NA |  | NA | NA | NA |
| rs13237474 | 7 | 135501469 | T/C | 0.03 | 1.115 | 0.214 | 2.00E-07 | 27.019 |  | -0.041 | 0.074 | 0.630 |  | -0.050 | 0.049 | 0.308 |
| rs1833219 | 2 | 67297409 | C/A | 0.72 | 0.278 | 0.062 | 8.00E-06 | 19.928 |  | 0.010 | 0.021 | 0.720 |  | 0.026 | 0.018 | 0.138 |
| rs1953021 | 9 | 12914396 | T/G | 0.71 | 0.300 | 0.061 | 1.00E-06 | 23.916 |  | 0.000 | 0.021 | 0.990 |  | 0.008 | 0.017 | 0.633 |
| rs2009196 | 17 | 76870581 | C/G | 0.23 | 0.344 | 0.072 | 2.00E-06 | 22.583 |  | 0.030 | 0.021 | 0.190 |  | -0.012 | 0.018 | 0.499 |
| rs2243407 | 8 | 11480457 | C/T | 0.65 | 0.285 | 0.063 | 6.00E-06 | 20.478 |  | 0.000 | 0.021 | 0.830 |  | 0.003 | 0.017 | 0.863 |
| rs2293335 | 1 | 204103618 | G/A | 0.3 | 0.300 | 0.066 | 5.00E-06 | 20.827 |  | 0.010 | 0.021 | 0.760 |  | 0.006 | 0.018 | 0.723 |
| rs263063 | 19 | 5022604 | T/C | 0.08 | 0.489 | 0.108 | 6.00E-06 | 20.478 |  | 0.030 | 0.036 | 0.460 |  | 0.019 | 0.028 | 0.495 |
| rs28455997 | 8 | 3076959 | T/C | 0.84 | 0.392 | 0.084 | 3.00E-06 | 21.805 |  | -0.030 | 0.027 | 0.350 |  | 0.023 | 0.023 | 0.316 |
| rs28643277 | 10 | 15600671 | A/C | 0.35 | 0.278 | 0.063 | 9.00E-06 | 19.703 |  | 0.020 | 0.020 | 0.410 |  | -0.015 | 0.017 | 0.379 |
| rs34069323 | 15 | 85187245 | C/T | 0.01 | 1.833 | 0.386 | 2.00E-06 | 22.583 |  | -0.157 | 0.324 | 0.630 |  | 0.018 | 0.082 | 0.822 |
| rs6497031 | 15 | 93713666 | A/G | 0.17 | 0.610 | 0.134 | 5.00E-06 | 20.827 |  | 0.010 | 0.026 | 0.630 |  | -0.029 | 0.022 | 0.177 |
| rs6890783^#^ | 5 | 172292306 | A/G | 0.01 | 1.864 | 0.408 | 5.00E-06 | 20.827 |  | NA | NA | NA |  | -0.008 | 0.075 | 0.917 |
| rs71455379 | 12 | 11558877 | G/C | 0.06 | 0.637 | 0.143 | 9.00E-06 | 19.703 |  | 0.191 | 0.108 | 0.074 |  | 0.008 | 0.032 | 0.793 |
| rs72899866 | 2 | 141226309 | A/T | 0.22 | 0.329 | 0.072 | 5.00E-06 | 20.827 |  | -0.020 | 0.021 | 0.360 |  | 0.020 | 0.019 | 0.287 |
| rs73389468 | 10 | 134295815 | C/T | 0.98 | 1.022 | 0.230 | 9.00E-06 | 19.703 |  | 0.062 | 0.095 | 0.500 |  | -0.131 | 0.060 | 0.031 |
| rs7819988 | 8 | 122210890 | T/C | 0.55 | 0.278 | 0.059 | 3.00E-06 | 21.805 |  | 0.020 | 0.020 | 0.370 |  | 0.000 | 0.016 | 0.982 |
| rs78411303 | 3 | 122356451 | A/G | 0.09 | 0.673 | 0.144 | 3.00E-06 | 21.805 |  | -0.041 | 0.050 | 0.450 |  | -0.004 | 0.032 | 0.908 |
| rs78797168 | 6 | 160459191 | A/G | 0.93 | 0.560 | 0.118 | 2.00E-06 | 22.583 |  | -0.010 | 0.037 | 0.870 |  | 0.000 | 0.030 | 0.987 |

Abbreviations: AgP: aggressive periodontitis; Chr: chromosome; CP: chronic periodontitis; EA/OA: effect allele/other allele; EAF: effect allele frequency; OA: osteoarthritis; PD: periodontitis; Pos: position according to GRCh37/hg19 genome assembly; RA: rheumatoid arthritis; SNP: single nucleotide polymorphism; SE: standard error of beta.

^*^rs4969455(T/C) was used as proxy SNP (linkage disequilibrium R^2^=1) of rs2672903(T/G) in the summary statistics of the outcome.

^#^Summary statistics for four SNPs(rs73155039, rs11633566, rs13145041, and rs6890783) were not available in the GWAS of RA or OA and were excluded as instrumental variables for periodontitis.

**Table S2.** Detailed information of instrumental variables utilized in the Mendelian Randomization analysis of rheumatoid arthritis on periodontitis.

| SNP | Chr | Pos | EA/OS | EAF | Association with RA | | | |  | Association with PD | | |
| --- | --- | --- | --- | --- | --- | --- | --- | --- | --- | --- | --- | --- |
|  |  |  |  |  | *β* | S.E. | *P*-value | F-statistic |  | *β* | S.E. | *P*-value |
| rs10175798 | 2 | 30449594 | A/G | 0.62 | 0.086 | 0.016 | 4.20E-08 | 30.054 |  | -0.019 | 0.019 | 0.309 |
| rs10774624 | 12 | 111833788 | G/A | 0.48 | 0.086 | 0.015 | 6.90E-09 | 33.562 |  | -0.043 | 0.019 | 0.022 |
| rs10790268 | 11 | 118729391 | G/A | 0.81 | 0.157 | 0.018 | 1.40E-17 | 72.846 |  | -0.011 | 0.023 | 0.639 |
| rs10985070 | 9 | 123636121 | C/A | 0.43 | 0.086 | 0.015 | 4.20E-09 | 34.528 |  | -0.028 | 0.018 | 0.122 |
| rs11574914 | 9 | 34710338 | A/G | 0.32 | 0.122 | 0.015 | 1.80E-15 | 63.271 |  | 0.025 | 0.019 | 0.191 |
| rs11889341 | 2 | 191943742 | T/C | 0.23 | 0.113 | 0.016 | 1.40E-12 | 50.182 |  | 0.035 | 0.021 | 0.097 |
| rs11933540 | 4 | 26120001 | C/T | 0.31 | 0.140 | 0.017 | 9.50E-17 | 69.068 |  | -0.006 | 0.025 | 0.802 |
| rs12140275 | 1 | 38633879 | A/T | 0.78 | 0.104 | 0.018 | 4.40E-09 | 34.437 |  | 0.003 | 0.021 | 0.878 |
| rs13330176 | 16 | 86019087 | A/T | 0.24 | 0.113 | 0.020 | 9.00E-09 | 33.045 |  | 0.069 | 0.027 | 0.011 |
| rs138193887 | 11 | 107967350 | A/G | 0.91 | 0.191 | 0.033 | 1.10E-08 | 32.655 |  | 0.038 | 0.035 | 0.280 |
| rs147622113 | 19 | 10771941 | C/T | 0.98 | 0.385 | 0.063 | 8.80E-10 | 37.573 |  | 0.023 | 0.069 | 0.736 |
| rs1516971 | 8 | 129542100 | T/C | 0.9 | 0.148 | 0.022 | 3.20E-11 | 44.049 |  | 0.032 | 0.027 | 0.238 |
| rs1571878 | 6 | 167540842 | C/T | 0.42 | 0.122 | 0.014 | 2.40E-18 | 76.328 |  | 0.006 | 0.018 | 0.743 |
| rs17264332 | 6 | 138005515 | G/A | 0.17 | 0.157 | 0.017 | 4.10E-20 | 84.369 |  | -0.025 | 0.022 | 0.257 |
| rs1858037 | 2 | 65598300 | T/A | 0.68 | 0.086 | 0.014 | 2.00E-09 | 35.973 |  | -0.011 | 0.019 | 0.551 |
| rs187786174 | 1 | 2523811 | G/A | 0.71 | 0.095 | 0.016 | 4.80E-09 | 34.268 |  | 0.032 | 0.021 | 0.128 |
| rs1893592 | 21 | 43855067 | A/C | 0.72 | 0.104 | 0.018 | 9.80E-09 | 32.879 |  | 0.020 | 0.020 | 0.308 |
| rs1950897 | 14 | 68760141 | T/C | 0.67 | 0.086 | 0.016 | 5.00E-08 | 29.716 |  | 0.009 | 0.020 | 0.636 |
| rs1980422 | 2 | 204610396 | C/T | 0.26 | 0.122 | 0.017 | 1.90E-13 | 54.104 |  | 0.012 | 0.020 | 0.567 |
| rs2105325 | 1 | 173349725 | C/A | 0.76 | 0.113 | 0.017 | 3.30E-11 | 43.989 |  | 0.012 | 0.020 | 0.561 |
| rs2233424 | 6 | 44233921 | T/C | 0.04 | 0.285 | 0.052 | 3.30E-08 | 30.521 |  | 0.027 | 0.045 | 0.552 |
| rs2234067 | 6 | 36355654 | C/A | 0.88 | 0.131 | 0.024 | 4.10E-08 | 30.101 |  | 0.011 | 0.027 | 0.692 |
| rs2301888 | 1 | 17672730 | G/A | 0.65 | 0.104 | 0.018 | 5.50E-09 | 34.003 |  | 0.000 | 0.019 | 0.994 |
| rs2451258 | 6 | 159506600 | T/C | 0.64 | 0.095 | 0.015 | 1.60E-10 | 40.901 |  | -0.002 | 0.019 | 0.918 |
| rs2476601 | 1 | 114377568 | A/G | 0.09 | 0.588 | 0.021 | 8.90E-170 | 771.381 |  | 0.009 | 0.029 | 0.772 |
| rs2561477 | 5 | 102608924 | G/A | 0.68 | 0.104 | 0.016 | 2.20E-11 | 44.783 |  | -0.028 | 0.019 | 0.137 |
| rs2664035 | 4 | 48220839 | A/G | 0.4 | 0.077 | 0.014 | 3.30E-08 | 30.522 |  | 0.029 | 0.018 | 0.109 |
| rs28411352 | 1 | 38278579 | T/C | 0.26 | 0.095 | 0.016 | 5.90E-09 | 33.866 |  | 0.011 | 0.022 | 0.609 |
| rs3087243 | 2 | 204738919 | G/A | 0.55 | 0.140 | 0.014 | 3.60E-22 | 93.736 |  | -0.017 | 0.018 | 0.338 |
| rs34536443 | 19 | 10463118 | G/C | 0.97 | 0.378 | 0.047 | 4.60E-16 | 65.958 |  | -0.001 | 0.056 | 0.985 |
| rs34695944 | 2 | 61124850 | C/T | 0.35 | 0.122 | 0.015 | 3.70E-16 | 66.388 |  | -0.041 | 0.018 | 0.025 |
| rs3778753 | 7 | 128580042 | G/A | 0.46 | 0.113 | 0.016 | 4.10E-12 | 48.074 |  | 0.035 | 0.019 | 0.063 |
| rs3806624 | 3 | 27764623 | G/A | 0.47 | 0.077 | 0.014 | 2.80E-08 | 30.840 |  | 0.028 | 0.019 | 0.142 |
| rs3824660 | 10 | 8104722 | C/T | 0.41 | 0.095 | 0.016 | 2.70E-09 | 35.388 |  | -0.018 | 0.018 | 0.316 |
| rs4239702 | 20 | 44749251 | C/T | 0.72 | 0.131 | 0.016 | 1.10E-16 | 68.779 |  | -0.001 | 0.020 | 0.944 |
| rs4272 | 7 | 92236829 | G/A | 0.23 | 0.095 | 0.017 | 1.20E-08 | 32.486 |  | 0.025 | 0.022 | 0.256 |
| rs4409785 | 11 | 95311422 | C/T | 0.19 | 0.113 | 0.019 | 3.60E-09 | 34.828 |  | 0.028 | 0.026 | 0.279 |
| rs4452313 | 3 | 17047032 | T/A | 0.3 | 0.104 | 0.016 | 5.20E-11 | 43.099 |  | -0.011 | 0.019 | 0.570 |
| rs4780401 | 16 | 11839326 | T/G | 0.6 | 0.086 | 0.015 | 8.70E-09 | 33.111 |  | -0.023 | 0.019 | 0.223 |
| rs59716545 | 17 | 38031857 | G/T | 0.48 | 0.086 | 0.014 | 6.00E-10 | 38.320 |  | -0.004 | 0.019 | 0.851 |
| rs624988 | 1 | 117263790 | T/C | 0.4 | 0.086 | 0.014 | 8.00E-10 | 37.759 |  | -0.007 | 0.018 | 0.715 |
| rs6715284 | 2 | 202154397 | G/C | 0.11 | 0.140 | 0.023 | 2.50E-09 | 35.538 |  | 0.010 | 0.029 | 0.728 |
| rs67250450 | 7 | 28174986 | T/C | 0.79 | 0.104 | 0.018 | 2.60E-09 | 35.462 |  | 0.038 | 0.022 | 0.075 |
| rs6732565 | 2 | 111607832 | A/G | 0.63 | 0.095 | 0.017 | 9.40E-09 | 32.960 |  | 0.031 | 0.018 | 0.085 |
| rs678347 | 8 | 102463602 | G/A | 0.23 | 0.095 | 0.016 | 7.30E-09 | 33.452 |  | -0.022 | 0.020 | 0.267 |
| rs706778 | 10 | 6098949 | T/C | 0.42 | 0.113 | 0.014 | 4.60E-15 | 61.423 |  | 0.016 | 0.018 | 0.362 |
| rs71508903 | 10 | 63779871 | T/C | 0.24 | 0.140 | 0.018 | 3.30E-15 | 62.077 |  | -0.005 | 0.023 | 0.838 |
| rs73081554 | 3 | 58302935 | T/C | 0.06 | 0.166 | 0.030 | 4.70E-08 | 29.836 |  | 0.018 | 0.038 | 0.644 |
| rs73194058 | 21 | 34764288 | C/A | 0.85 | 0.122 | 0.022 | 2.60E-08 | 30.984 |  | 0.009 | 0.027 | 0.735 |
| rs773125 | 12 | 56394954 | A/G | 0.62 | 0.086 | 0.015 | 2.10E-08 | 31.399 |  | 0.029 | 0.019 | 0.123 |
| rs7731626 | 5 | 55444683 | G/A | 0.63 | 0.191 | 0.019 | 8.20E-23 | 96.663 |  | -0.050 | 0.023 | 0.025 |
| rs7752903 | 6 | 138227364 | G/T | 0.02 | 0.344 | 0.037 | 1.70E-20 | 86.110 |  | -0.069 | 0.054 | 0.200 |
| rs8026898 | 15 | 69991417 | A/G | 0.29 | 0.140 | 0.016 | 5.90E-18 | 74.552 |  | -0.002 | 0.020 | 0.927 |
| rs8032939 | 15 | 38834033 | C/T | 0.25 | 0.122 | 0.016 | 3.20E-14 | 57.606 |  | -0.012 | 0.020 | 0.554 |
| rs8083786 | 18 | 12881361 | G/A | 0.15 | 0.113 | 0.020 | 8.40E-09 | 33.179 |  | 0.009 | 0.025 | 0.722 |
| rs8133843 | 21 | 36738242 | A/G | 0.63 | 0.086 | 0.015 | 2.00E-08 | 31.494 |  | 0.012 | 0.019 | 0.511 |
| rs909685 | 22 | 39747671 | A/T | 0.31 | 0.104 | 0.015 | 6.40E-12 | 47.202 |  | 0.005 | 0.020 | 0.816 |
| rs9372120 | 6 | 106667535 | G/T | 0.19 | 0.095 | 0.017 | 3.80E-08 | 30.248 |  | -0.015 | 0.023 | 0.506 |
| rs947474 | 10 | 6390450 | A/G | 0.82 | 0.113 | 0.018 | 3.30E-10 | 39.487 |  | 0.002 | 0.022 | 0.936 |
| rs9603616 | 13 | 40368069 | C/T | 0.67 | 0.104 | 0.016 | 2.80E-11 | 44.311 |  | -0.008 | 0.019 | 0.676 |
| rs9653442 | 2 | 100825367 | C/T | 0.46 | 0.113 | 0.015 | 9.80E-15 | 59.934 |  | 0.010 | 0.018 | 0.560 |
| rs968567 | 11 | 61595564 | C/T | 0.83 | 0.113 | 0.020 | 1.80E-08 | 31.698 |  | 0.015 | 0.023 | 0.525 |
| rs9826828^#^ | 3 | 136402060 | A/G | 0.02 | 0.365 | 0.059 | 8.70E-10 | 37.595 |  | NA | NA | NA |
| rs998731 | 8 | 81095395 | T/C | 0.49 | 0.086 | 0.015 | 6.60E-09 | 33.648 |  | -0.030 | 0.018 | 0.091 |

Abbreviations: Chr: chromosome; EA/OA: effect allele/other allele; EAF: effect allele frequency; PD: periodontitis; Pos: position according to GRCh37/hg19 genome assembly; RA: rheumatoid arthritis; SE: standard error of beta; SNP: single nucleotide polymorphism;.

^#^Summary statistic for SNP rs9826828 was not available in the GWAS of PD , and so was excluded as instrumental variable for RA.

**Table S3.** Detailed information of instrumental variables utilized in the Mendelian Randomization analysis of osteoarthritis on periodontitis.

| SNP | Chr | Pos | EA/OS | EAF | Association with OA | | | |  | Association with PD | | |
| --- | --- | --- | --- | --- | --- | --- | --- | --- | --- | --- | --- | --- |
|  |  |  |  |  | *β* | S.E. | *P*-value | F-statistic |  | *β* | S.E. | *P*-value |
| rs1044369 | 20 | 44987318 | G/C | 0.31 | -0.077 | 0.017 | 7.23E-06 | 20.002 |  | 0.011 | 0.028 | 0.703 |
| rs112354755 | 3 | 162868729 | T/C | 0.06 | -0.173 | 0.035 | 6.41E-07 | 23.981 |  | -0.006 | 0.066 | 0.926 |
| rs11620787 | 14 | 35096198 | A/G | 0.18 | 0.097 | 0.020 | 1.72E-06 | 23.141 |  | -0.026 | 0.024 | 0.285 |
| rs11686353 | 2 | 236561934 | C/A | 0.26 | -0.082 | 0.019 | 8.56E-06 | 19.630 |  | -0.017 | 0.040 | 0.672 |
| rs116882138 | 9 | 27313557 | A/G | 0.02 | 0.262 | 0.057 | 6.05E-06 | 21.385 |  | -0.147 | 0.078 | 0.059 |
| rs12193876 | 6 | 89260265 | T/C | 0.18 | -0.100 | 0.021 | 2.54E-06 | 21.820 |  | 0.027 | 0.023 | 0.244 |
| rs12731575 | 1 | 103343274 | C/T | 0.05 | -0.166 | 0.037 | 5.15E-06 | 20.112 |  | 0.032 | 0.039 | 0.416 |
| rs143383 | 20 | 34025983 | G/A | 0.36 | -0.084 | 0.017 | 3.53E-07 | 25.791 |  | 0.011 | 0.019 | 0.549 |
| rs143983158 | 20 | 21616475 | A/G | 0.02 | -0.277 | 0.064 | 7.29E-06 | 18.715 |  | 0.042 | 0.070 | 0.547 |
| rs17712916 | 12 | 64607264 | A/G | 0.06 | 0.149 | 0.033 | 7.29E-06 | 20.594 |  | -0.054 | 0.055 | 0.320 |
| rs1886647 | 1 | 85354563 | A/G | 0.28 | -0.084 | 0.018 | 3.43E-06 | 21.371 |  | 0.006 | 0.021 | 0.783 |
| rs2231495 | 22 | 17669306 | C/T | 0.33 | 0.078 | 0.017 | 3.01E-06 | 21.910 |  | -0.007 | 0.019 | 0.720 |
| rs2820436 | 1 | 219640680 | C/A | 0.66 | -0.075 | 0.017 | 6.45E-06 | 20.430 |  | -0.035 | 0.019 | 0.068 |
| rs3851225 | 6 | 111825132 | G/A | 0.57 | -0.071 | 0.016 | 8.49E-06 | 19.853 |  | 0.005 | 0.018 | 0.772 |
| rs4233567 | 2 | 144272376 | T/C | 0.36 | -0.082 | 0.017 | 1.26E-06 | 23.352 |  | 0.010 | 0.020 | 0.602 |
| rs4380275 | 2 | 773278 | T/C | 0.65 | 0.074 | 0.017 | 7.95E-06 | 19.847 |  | -0.014 | 0.019 | 0.454 |
| rs56075670 | 1 | 18418325 | A/G | 0.01 | -0.356 | 0.081 | 4.28E-06 | 19.395 |  | 0.039 | 0.114 | 0.731 |
| rs62374753 | 5 | 124768796 | A/G | 0.03 | 0.211 | 0.046 | 5.99E-06 | 21.253 |  | -0.005 | 0.057 | 0.929 |
| rs6546174 | 2 | 65935393 | T/C | 0.53 | -0.087 | 0.018 | 1.81E-06 | 22.792 |  | -0.002 | 0.019 | 0.920 |
| rs6977416 | 7 | 150542711 | A/G | 0.33 | -0.076 | 0.017 | 7.84E-06 | 19.859 |  | -0.016 | 0.019 | 0.408 |
| rs72718147 | 9 | 25036933 | A/G | 0.16 | -0.107 | 0.024 | 5.71E-06 | 20.223 |  | 4.00E-04 | 0.035 | 0.992 |
| rs73080980 | 3 | 50196533 | T/C | 0.21 | -0.095 | 0.020 | 1.99E-06 | 22.331 |  | -0.029 | 0.023 | 0.208 |
| rs75944996 | 9 | 72409180 | G/A | 0.05 | -0.175 | 0.040 | 6.63E-06 | 19.618 |  | 0.063 | 0.051 | 0.215 |
| rs7714497 | 5 | 112006948 | G/A | 0.18 | -0.092 | 0.021 | 9.07E-06 | 19.452 |  | 0.032 | 0.023 | 0.156 |
| rs78115154 | 5 | 152899101 | C/T | 0.04 | -0.197 | 0.044 | 3.97E-06 | 20.450 |  | 0.087 | 0.060 | 0.150 |
| rs9342489 | 6 | 66344801 | G/A | 0.03 | -0.221 | 0.048 | 2.92E-06 | 20.868 |  | 0.030 | 0.051 | 0.562 |

Abbreviations: Chr: chromosome; EA/OA: effect allele/other allele; EAF: effect allele frequency; OA: osteoarthritis; PD: periodontitis; Pos: position according to GRCh37/hg19 genome assembly; SE: standard error of beta; SNP: single nucleotide polymorphism;.

**Table S4.** Assessing directional pleiotropy through MR-Egger intercept and MR-PRESSO test.

| **Exposure** | **Outcome** | **MR-Egger intercept** | | |  | **MR-PRESSO global test** | |
| --- | --- | --- | --- | --- | --- | --- | --- |
|  |  | **Intercept** | **SE** | **Pval** |  | **RSS_obs_** | ***P*-value** |
| PD | RA | 0.015 | 0.021 | 0.47 |  | 28.22 | 0.08 |
|  | OA | 0.012 | 0.011 | 0.30 |  | 16.79 | 0.62 |
| AgP | RA | 0.041 | 0.040 | 0.31 |  | 13.20 | 0.46 |
|  | OA | 0.031 | 0.031 | 0.35 |  | 15.67 | 0.36 |
| CP | RA | 0.011 | 0.010 | 0.50 |  | 15.11 | 0.81 |
|  | OA | 0.013 | 0.011 | 0.24 |  | 19.19 | 0.60 |
| RA | PD | 0.003 | 0.007 | 0.66 |  | 76.06 | 0.14 |
| OA | PD | 0.026 | 0.015 | 0.09 |  | 19.09 | 0.87 |


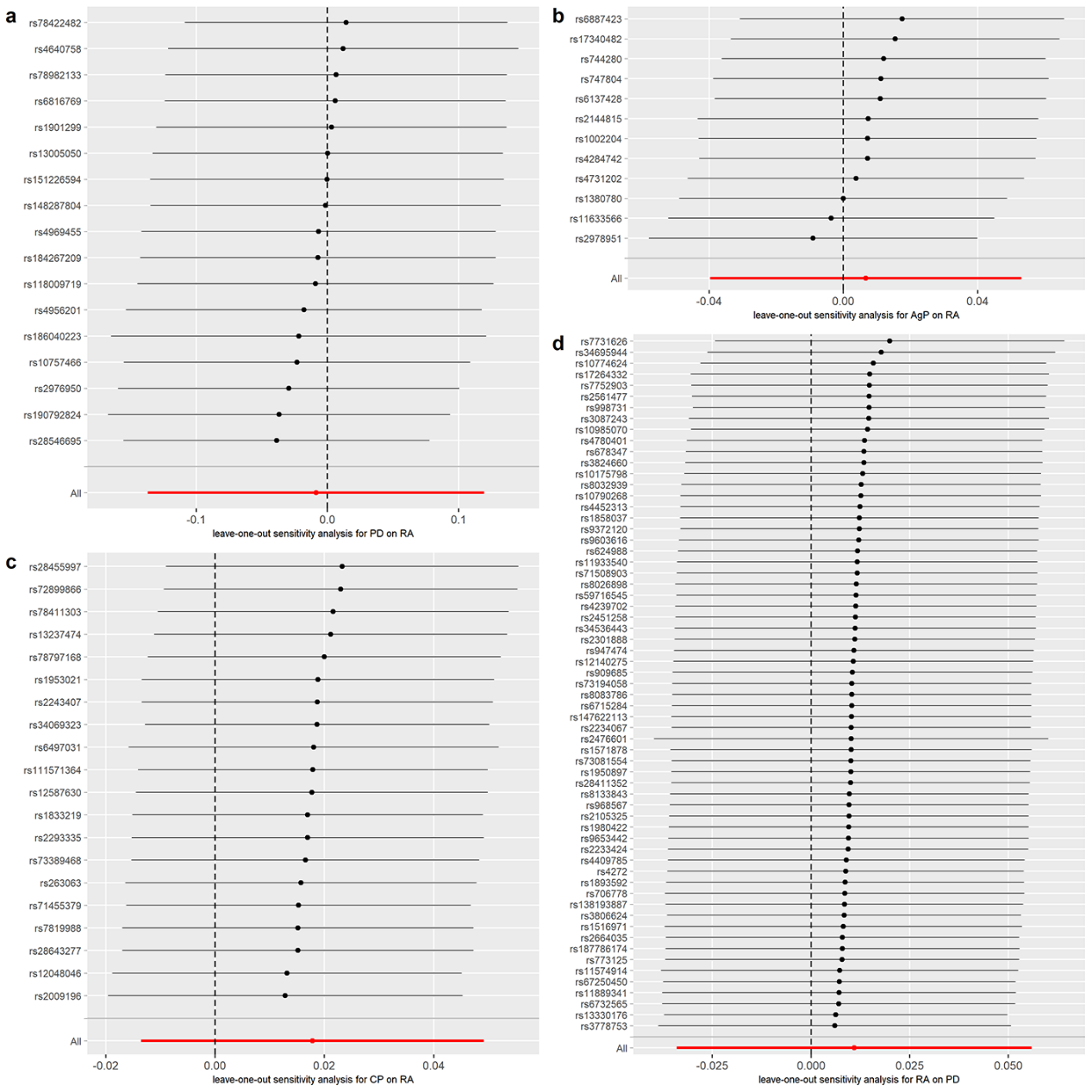


**Figure S1.** Leave-one-out plot of the causal relationships between RA and periodontitis phenotypes. (a) MR estimates for PD on RA. (b) MR estimates for AgP on RA. (c) MR estimates for CP on RA. (d) MR estimates for RA on PD. The leave-one-out plot visualized how the causal estimates (point with horizontal line) between RA and periodontitis phenotypes were influenced by the removal of single variant.


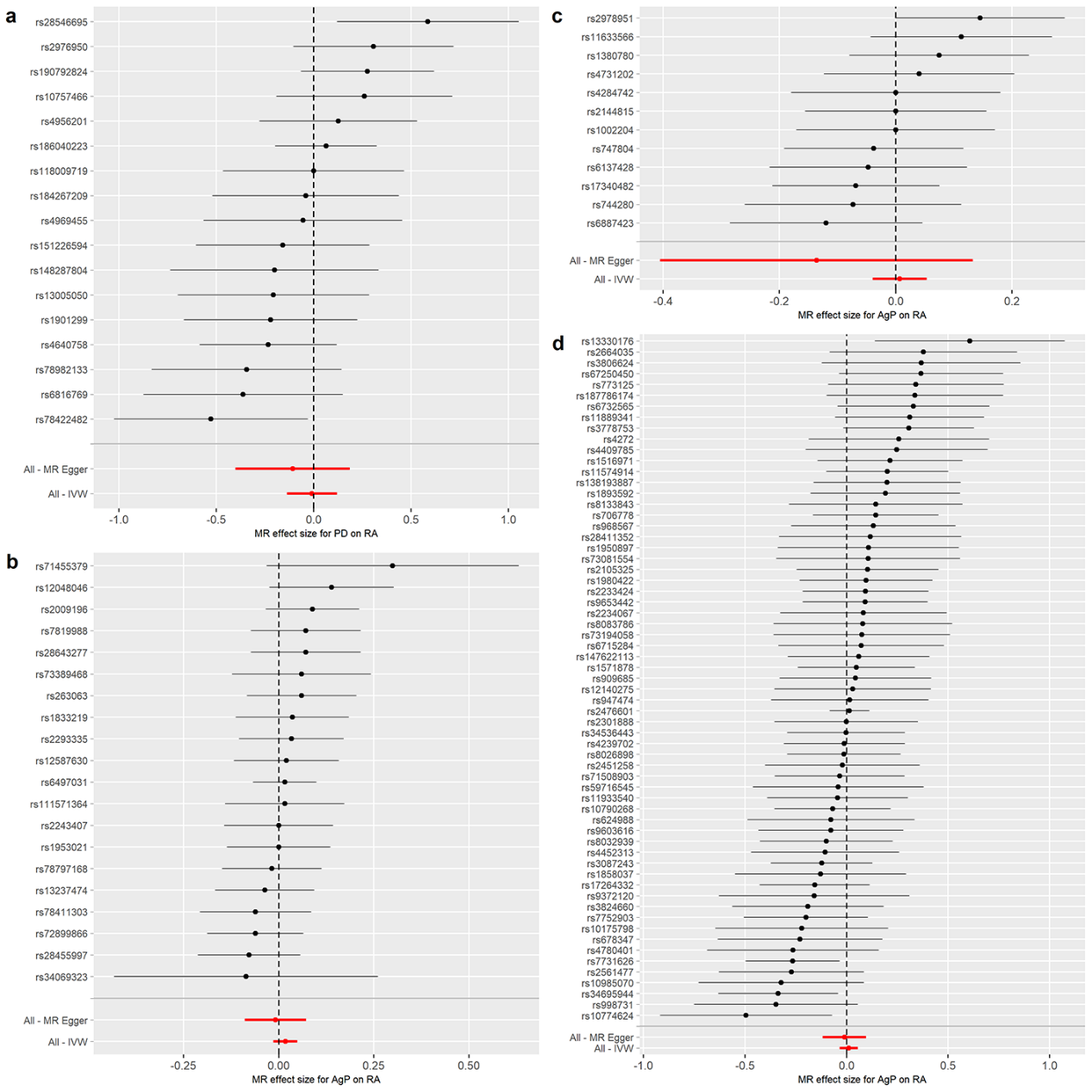


**Figure S2.** Forrest plot of the causal relationships between RA and periodontitis phenotypes. (a) MR estimates for PD on RA. (b) MR estimates for AgP on RA. (c) MR estimates for CP on RA. (d) MR estimates for RA on PD.


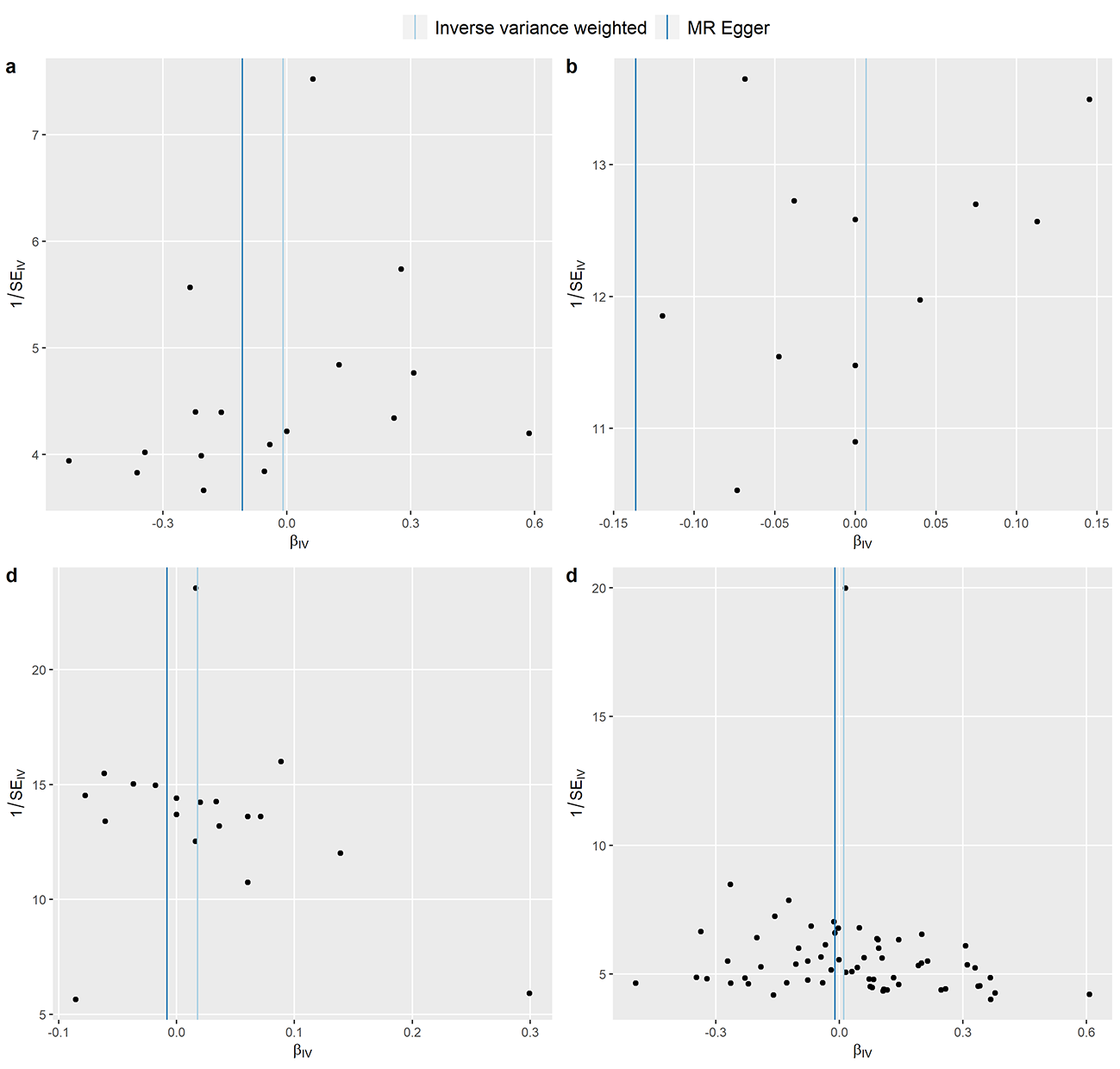


**Figure S3.** Funnel plot of the causal relationships between RA and periodontitis phenotypes. (a) MR estimates for PD on RA. (b) MR estimates for AgP on RA. (c) MR estimates for CP on RA. (d) MR estimates for RA on PD. The funnel plot illustrated the overall symmetry of causal estimates across all instrumental variables.


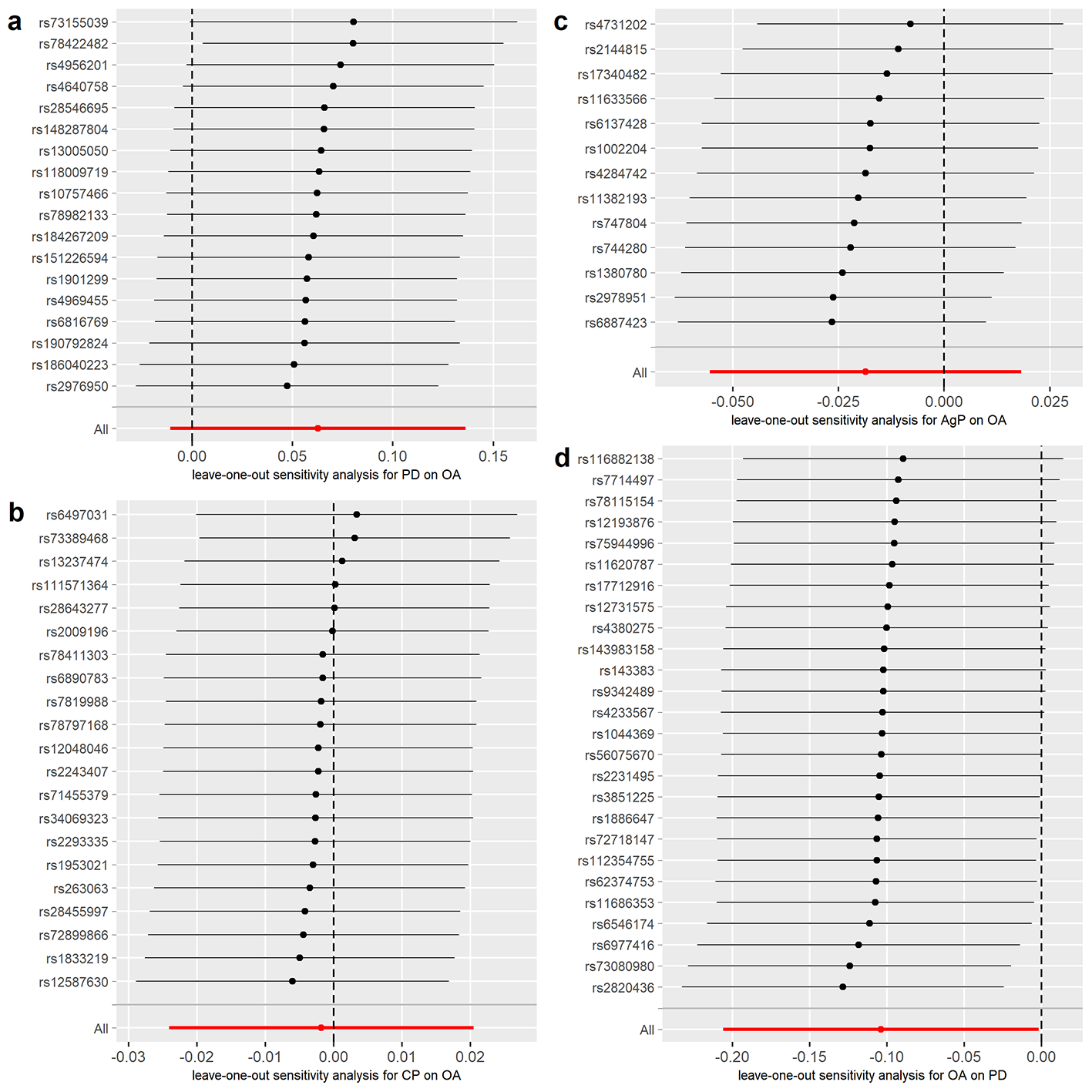


**Figure S4.** Leave-one-out plot of the causal relationships between OA and periodontitis phenotypes. (a) MR estimates for periodontitis on OA. (b) MR estimates for aggressive periodontitis on OA. (c) MR estimates for chronic periodontitis on OA. (d) MR estimates for OA on periodontitis. The leave-one-out plot visualized how the causal estimates (point with horizontal line) between OA and periodontitis phenotypes were influenced by the removal of single variant.


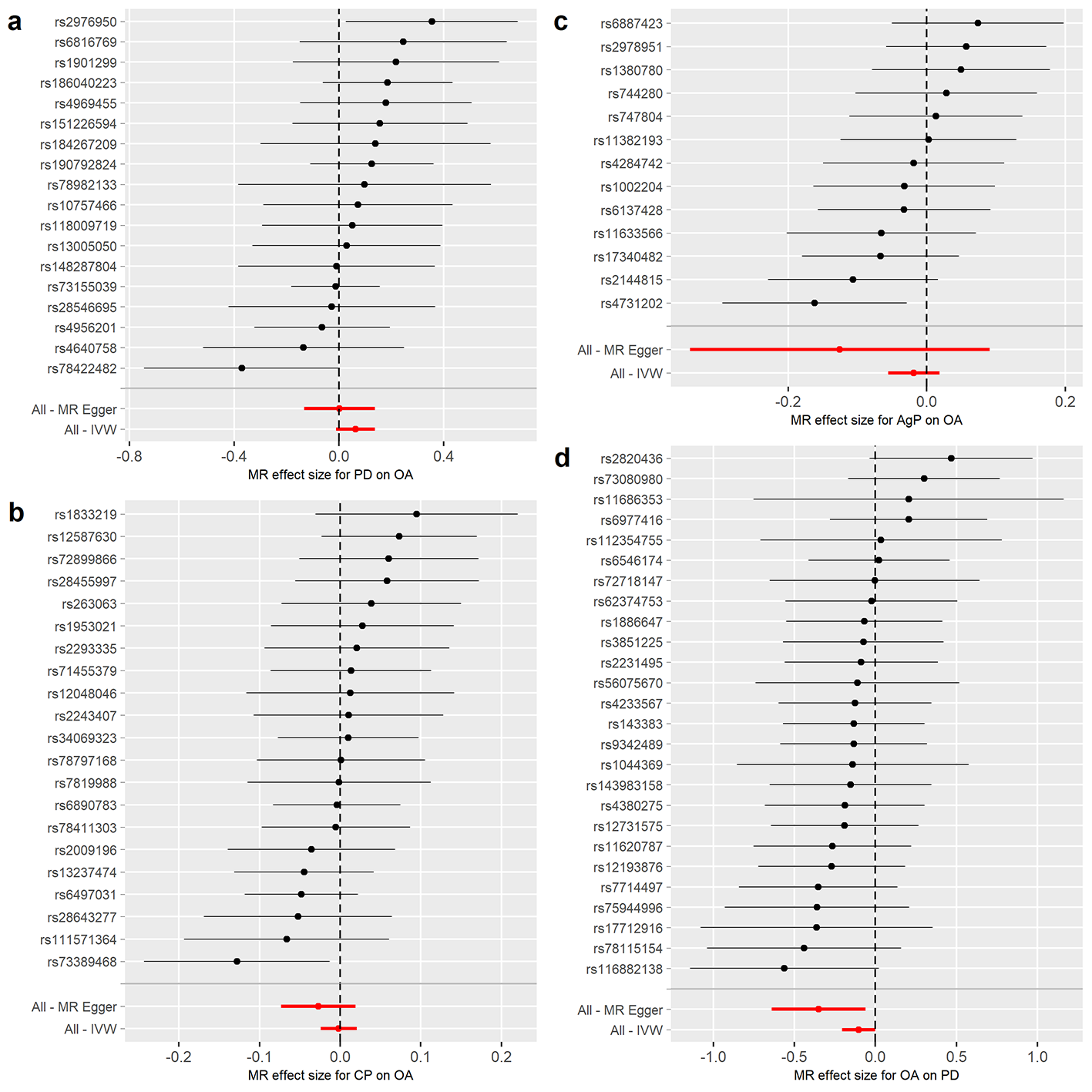
**Figure S5.** Forrest plot of the causal relationships between OA and periodontitis phenotypes. (a) MR estimates for periodontitis on OA. (b) MR estimates for aggressive periodontitis on OA. (c) MR estimates for chronic periodontitis on OA.


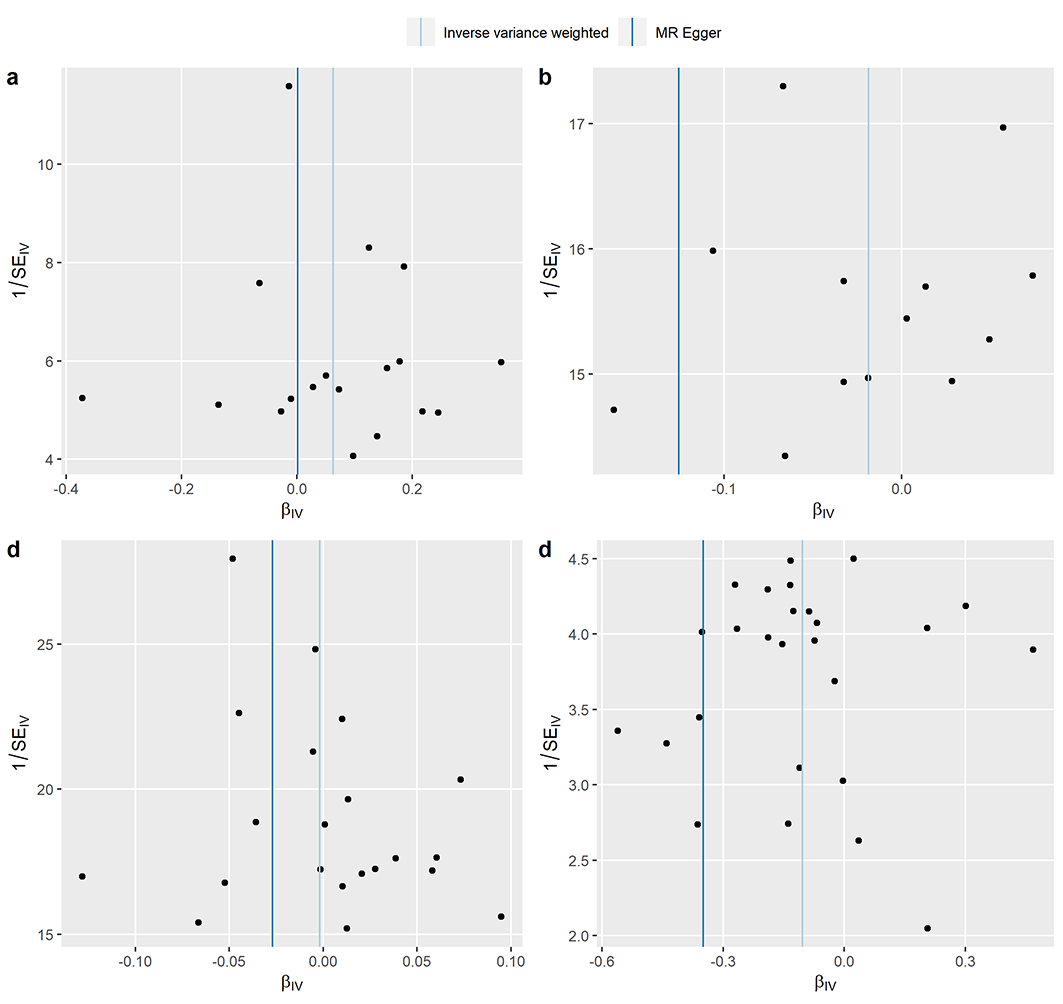


**Figure S6.** Funnel plot of the causal relationships between OA and periodontitis phenotypes. (a) MR estimates for periodontitis on OA. (b) MR estimates for aggressive periodontitis on OA. (c) MR estimates for chronic periodontitis on OA. (d) MR estimates for OA on periodontitis. The funnel plot illustrated the overall symmetry of causal estimates across all instrumental variables.
